# Mobilising Knowledge for General Practice Decarbonisation: Maximising Impact Through a Multi‐Stakeholder Workshop

**DOI:** 10.1111/hex.70477

**Published:** 2025-11-03

**Authors:** Ana Raquel Nunes, Olivia Geddes, Florence Karaba, Abi Eccles, Helen Twohig, Helen Atherton, Frederik Dahlmann, Michael Gregg, Rachel Spencer, Jeremy Dale

**Affiliations:** ^1^ Warwick Medical School University of Warwick Coventry UK; ^2^ Nuffield Department of Primary Care Health Sciences University of Oxford Oxford UK; ^3^ School of Medicine Keele University Keele UK; ^4^ School of Primary Care University of Southampton Southampton UK; ^5^ Warwick Business School University of Warwick Coventry UK

**Keywords:** climate change, decarbonisation, environmental sustainability, general practice, greenhouse gas emissions, implementation, knowledge mobilisation, net zero, research impact

## Abstract

**Background:**

This manuscript explores how knowledge mobilisation (KMb), through a multi‐stakeholder workshop, can advance decarbonisation in general practice by interpreting findings from the GPNET‐0 study, which includes a systematic review, a GP survey and longitudinal case studies. The KMb workshop serves as a platform to interpret and disseminate its findings, thereby informing the development and refinement of outputs (i.e., Policy Brief and Factsheet) and the tailoring of audience‐specific dissemination strategies.

**Methods:**

Fifteen stakeholders were selected from key groups across the United Kingdom, including public representatives, policymakers and general practitioners. The workshop was structured with presentations, group discussions and interactive activities to encourage targeted dialogue. Pre‐workshop materials, including a Policy Brief and Factsheet, were shared with participants to promote informed discussions. The workshop's design ensured that stakeholder input was actively integrated into the development and refinement of outputs.

**Findings:**

The KMb workshop provided valuable insights and views into the barriers, opportunities and priorities for general practice decarbonisation. The analysis identified four overarching views: ‘bridging knowledge and resource gaps’, ‘organisational and cultural barriers’, ‘enhancing public engagement and messaging’, and ‘policy and leadership as drivers of change’.

**Conclusions:**

The KMb effectively engaged stakeholders in reflecting on decarbonisation challenges and opportunities, informing the development of targeted dissemination strategies. The KMb workshop demonstrated the critical role of stakeholder engagement in refining outputs for maximal impact of research findings and outputs.

**Patient or Public Contribution:**

Patients and members of the public were closely involved throughout the study. Two lay representatives served as co‐investigators, and a nine‐member PPI panel provided input across all stages, including study design, development of the Policy Brief and Factsheet, and the knowledge mobilisation workshop, and one lay representative is a co‐author of this article.

## 1 Introduction

The transition to net zero healthcare systems is a crucial element of the global response to climate change and its associated health impacts [1]. Healthcare systems are major contributors to carbon emissions [2, 3], making their decarbonisation a vital step towards sustainability [4]. Achieving net zero in healthcare requires coordinated efforts to translate scientific research findings into actionable solutions that can be readily implemented, while simultaneously promoting a culture of sustainability [5].

In this context, knowledge mobilisation (KMb) is seen as a key mechanism to drive impact by actively bringing together stakeholders throughout the research cycle to share, respond to and act upon research plans and findings [6]. We acknowledge that several terms, such as knowledge translation, integrated knowledge translation and KMb, are used interchangeably in this field. Here, we adopted the term ‘knowledge mobilisation’ to emphasise the active, iterative and co‐productive processes of engagement beyond linear translation of evidence. While ‘knowledge translation’ is broadly used, ‘mobilisation’ better reflects the collaborative and participatory ethos underpinning this study. KMb in healthcare is increasingly acknowledged as a crucial mechanism for bridging the gap between research, policy and practice by fostering collaboration among researchers, policymakers, practitioners, service users and communities [7, 8]. Such collaborative approaches enhance the applicability of research, ensuring that evidence is actively mobilised, exchanged and implemented to foster meaningful change [9, 10]. Co‐production of knowledge is particularly valued for its capacity to generate sustained research impacts and drive systemic transformation [11, 12].

Effective KMb for greener healthcare requires the creation of collaborative platforms that empower stakeholders in addition to standard dissemination so that they may adopt and scale decarbonisation practices in their respective domains [13, 14]. Engagement, mobilisation and exchange are pivotal in aligning diverse stakeholders, ranging from healthcare professionals to policymakers, commissioners, patients, public and community groups around shared sustainability goals [14, 15]. However, achieving this alignment is not merely a matter of dissemination, it requires actively convincing stakeholders of the importance of decarbonisation, as agreement with its principles is not a given. Overcoming scepticism, competing priorities and varying levels of awareness of net zero is essential to fostering meaningful commitment and enabling actionable change.

The National Institute of Health Research (NIHR) funded GPNET‐0 study was designed to examine the implementation of decarbonisation strategies in general practice, identifying key barriers, enablers and pathways towards achieving net zero [16]. The study began in September 2023 and is guided by a central research question: *‘How do institutional, organisational, professional, and patient factors influence the implementation and sustainability of actions to mitigate the greenhouse gas emissions associated with general practice?’*. Its overarching aims are to:

1. Understand how decarbonisation actions are being implemented in general practice to support the NHS's net zero ambitions and
2. To generate actionable recommendations for enhancing and accelerating their implementation and long‐term sustainability [16].

As part of this study, a systematic review was conducted to synthesise existing evidence on decarbonisation actions in general practice, identifying effective strategies, knowledge gaps and opportunities for informing policy and practice [17, 18]. Secondly, a survey of GP staff in three Integrated Care Systems (ICSs) in England found strong support for decarbonisation but identified gaps in training, resources and NHS support. In England, ICSs are partnerships of organisations that plan and deliver health and care services regionally. Practices with a designated lead undertook more actions and engaged more effectively. Key barriers included workload pressures, funding constraints and insufficient leadership, with challenges consistent across settings [19].

Additionally, the study incorporates longitudinal case studies to explore and compare the real‐world experiences of general practices implementing decarbonisation actions, capturing variations in context, engagement and system‐wide impact over time (12 months) [16]. The case studies were conducted in 12 diverse general practices recruited from those who had participated in the survey (detailed findings reported elsewhere). The three ICSs were selected to reflect geographical diversity (urban, semi‐urban and rural areas) and varying degrees of decarbonisation interest. The 12 general practices included in the case studies were drawn from survey respondents within these ICSs. The KMb workshop involved stakeholders from across the United Kingdom.

General practices, as a cornerstone of healthcare delivery, represent a critical yet often overlooked component in the broader decarbonisation agenda [3]. Primary care settings such as general practices account for a significant proportion of healthcare activity and, consequently, its carbon footprint [3]. However, the level of awareness, resources and support available to general practices to implement decarbonisation strategies remains limited [20]. The mobilisation of knowledge in this area is crucial to bridging these gaps.

Effective KMb in the context of decarbonisation for general practice involves not only raising awareness about sustainability practices and the need for these but also providing healthcare professionals with the tools, resources and organisational support needed to integrate these practices into everyday care delivery. Integrating decarbonisation efforts require flexible and responsive strategies that address the challenges general practice already face, including a current lack of clear guidance [5]. Thus, exploring KMb in decarbonisation in general practice is vital for ensuring that general practice is equipped to play an active role in achieving net zero. Embedding KMb successfully requires not only effective communication but also mechanisms for co‐production, stakeholder engagement and ongoing support to facilitate sustained change [8, 10].

Our aim, in the work reported here, was to explore the role of KMb in advancing general practice decarbonisation by examining the process, outcomes and insights generated from a collaborative KMb workshop. Herein we describe: (1) the structure and facilitation of the KMb workshop, (2) an assessment of its effectiveness in engaging diverse stakeholders in reflecting on the GPNET‐0 study findings and informing dissemination strategies, and (3) key views emerging from discussions to enhance understanding of decarbonisation in general practice. For the purposes of this article, ‘stakeholders’ refers to individuals and representatives from organisations directly or indirectly involved in, affected by or responsible for implementing decarbonisation actions in general practice. This includes but is not limited to general practice professionals, policymakers, NHS administrators, sustainability leads and patient representatives.

## 2 Methods

### 2.1 Stakeholders

Stakeholders were identified through targeted invitations to individuals known to have professional or experiential engagement with sustainability in primary care, identified via networks, groups and professional associations. We selected stakeholders from across the United Kingdom through a targeted selection process, ensuring the inclusion of key individuals with a vested interest and understanding of sustainability in general practice decarbonisation. These stakeholders, drawn from relevant groups such as public representatives, policymakers and commissioners, and general practitioners, were purposefully selected. Invitations were sent via email, providing potential stakeholders with an overview of the workshop's purpose and its role in shaping dissemination and implementation strategies for the GPNET‐0 study. The invitation emphasised the collaborative nature of the workshop and the opportunity for stakeholders to contribute their expertise and perspectives.

Fifteen stakeholders were purposively selected to ensure balanced representation of key perspectives. This sample size was chosen to maintain manageability for in‐depth discussion within a 90‐min online workshop. Efforts were made to include diverse voices, including those potentially less engaged with sustainability agenda, though participation ultimately reflected voluntary interest.

### 2.2 Workshop Structure

Designed with input from all members of the GPNET‐0 study research team, including public representatives, the workshop sought to promote collaboration among stakeholders to deepen understanding of the challenges surrounding general practice decarbonisation and to co‐develop effective dissemination and implementation strategies. The workshop was held online to minimise the carbon impact of the event, aligning with the study's broader goal of promoting decarbonisation in general practice (see Table 1). This 90‐min online format was chosen to balance engagement with minimal carbon impact. The discussion topics were structured around the emerging findings from the GPNET‐0 study, particularly: a systematic review that synthesised existing evidence on decarbonisation actions in general practice, highlighting effective strategies, gaps in knowledge and opportunities for policy and practice [17, 18]; a general practice survey asking about attitudes, barriers and enablers to decarbonisation in primary care, as well as current initiatives, resource availability and levels of engagement [19]; and emerging findings from ongoing longitudinal case study workshops and focus groups. Findings informed and helped refine the KMb workshop's scope and format.

**Table 1:** KMb workshop key details.

Venue	Online via MS Teams
Duration	90 min
Structure	Two panel sessions, two panel discussions and two facilitated small group discussions
Number of stakeholders	15 stakeholders
Payment/compensation	PPI stakeholders received payment for their time (i.e., NIHR standard rates)

The KMb workshop's structure followed a programme aimed at fostering active engagement and meaningful discussions through interactive group activities and discussions that encouraged participation and exchange of diverse perspectives (see Supporting Information 1). KMb workshop materials, including the study's Policy Brief #1 (Supporting Information 2) and Factsheet #1 (Supporting Information 3), were shared with stakeholders before the workshop. These resources were intended to facilitate informed engagement and meaningful discussions during the workshop. Members of the GPNET‐0 study's Patient and Public Involvement (PPI) panel provided feedback on drafts of the Policy Brief and Factsheet before they were shared ensuring clarity, relevance and accessibility for all stakeholders.

The workshop started with a presentation session outlining the study's key findings, introducing the Policy Brief and Factsheet. Stakeholders then engaged in small group discussions, structured into three groups according to their roles: (1) public representatives; (2) policymakers and commissioners; and (3) general practitioners, to ensure targeted and relevant dialogue. Each group included a facilitator and rapporteur from the study team. Discussions were guided by a set of prompts designed to explore which findings were most significant from their perspectives, whether there were any unexpected results, and what additional insights or questions should be considered. Rapporteurs summarised key points, which were then presented back in a discussion session. After a short break, a presentation session outlined current target audiences for the study findings and dissemination strategies. This was followed by another set of small group discussions, which focused on refining key messages and optimising dissemination approaches for the Policy Brief and Factsheet. The discussions explored how best to communicate findings, enhance impact and effectively reach relevant audiences. Again, rapporteurs summarised the discussions, and these insights were shared in a final discussion session.

Grouping stakeholders by role facilitated focused discussion on domain‐specific challenges. However, in plenary sessions, cross‐group sharing enabled reflection across perspectives, ensuring that insights from each group informed collective interpretation and synthesis.

The KMb workshop concluded with a summary of key outcomes and next steps, including plans for follow‐up engagement and a commitment to sharing a ‘You Said, We Did’ document based on stakeholder contributions.

KMb was not confined to this workshop; it was integrated across all stages of the GPNET‐0 Study, informing the research design, stakeholder engagement and dissemination. Although no single KMb framework was formally adopted, the workshop and subsequent dissemination activities were conceptually guided by the NIHR Knowledge Mobilisation Framework, which emphasises co‐production, feedback loops and shared learning between researchers and end users.

### 2.3 Ethical Considerations

All participants provided informed consent for video recording and anonymised use of quotes.

### 2.4 Workshop Evaluation

An online evaluation form was distributed to capture feedback from stakeholders on their overall experience, the content and delivery of the workshop, and suggestions for improvement to inform future workshops. The form comprised a total of six questions: four closed‐ended questions and two open‐ended questions, allowing for qualitative input. Responses were submitted anonymously to encourage honest and constructive feedback (see Supporting Information 4).

### 2.5 Analysis

The small group discussions were video recorded, and transcripts were generated. For the purposes of this manuscript, thematic summaries of each session and a thematic overview of the discussions were produced by the lead author (A.R.N.). The analysis process followed the principles of qualitative data analysis [21]. The analysis advanced through sequential stages using an inductive approach to capture unanticipated insights [21]. Themes were iteratively refined and codes grouped.

### 2.6 PPI

Patients and public were actively involved in the development of the overall study, as well as the Policy Brief and Factsheet. They contributed to the design of the KMb workshop, participated in its activities and provided valuable insights. Additionally, one representative is a co‐author of this paper. All PPI activities were conducted in accordance with national guidelines [22]. PPI co‐authors reviewed the original draft article and subsequent versions.

## 3 Results

The KMb workshop brought together 15 stakeholders, including public representatives, policymakers and commissioners, and general practitioners (see Table 2).

**Table 2:** KMb workshop stakeholders, facilitators, rapporteurs and note takers by role.

Group 1	Group 2	Group 3
3 PPI representatives	7 policymakers and commissioners	5 general practitioners

The analysis identified four overarching themes: ‘bridging knowledge and resource gaps’, ‘organisational and cultural barriers’, ‘enhancing public engagement and messaging’, and ‘policy and leadership as drivers of change’.

### 3.1 Bridging Knowledge and Resource Gaps

Stakeholders reported that many general practices were unaware of tools, funding streams and educational resources designed to aid decarbonisation. This gap was exacerbated by confusion over the specific purposes of resources, with some stakeholders highlighting the need to better understand what each resource does, how to use it and the distinctions between different tools.

‘[…] it is a bit surprising how many are unaware or have just heard of, but there is a small percentage that have used those ten resources’.

Policymaker

Stakeholders also noted that the low response rate in the GPNET‐0 study general practice survey (in total, 34% of general practices in the areas studied) [19] could indicate that interest in and active engagement with sustainability is significantly lower than reflected in the findings, as those already interested in the topic may have been more likely to respond.

Stakeholders highlighted the need to expand outreach and raise awareness across less engaged general practices. Simplified, consolidated and accessible resources were identified as critical to addressing these gaps. Stakeholders emphasised the importance of proactive KMb to ensure resources are effectively disseminated to diverse audiences, including clinical and non‐clinical general practice staff and patients.

‘I think that sitting at the ICB, it is things around how we start to bring different things together. […] people being unaware of recycling…. We are doing some of that already, but it is the wider training and education for me’.

Commissioner

The discussions also highlighted the need for tailored resources that consider the specific contexts and constraints faced by individual general practices. Stakeholders suggested that educational materials could include case studies and practical examples to inspire action and provide clarity on how to implement decarbonisation initiatives at the practice level.

‘They are not really aware of (resources), so obviously more training and more knowledge of it would be beneficial for staff’.

PPI representative

### 3.2 Practice‐Level Barriers to Implementation

Stakeholders highlighted the need to integrate decarbonisation into routine or everyday operations and decision‐making processes rather than being seen as an additional responsibility. For some, this was viewed as requiring a shift in organisational culture. Hence, leadership within general practices, particularly from senior staff, such as practice managers and GPs, was deemed crucial in championing sustainability and engaging the wider team. Stakeholders noted that successful integration depends on recognising sustainability as a shared responsibility, involving not just clinicians but also non‐clinical staff such as receptionists and administrative teams.

‘Actually it should be part of every day’.

Commissioner

Systemic barriers, such as workload pressures and the lack of ring‐fenced time necessary to focus on decarbonisation efforts, were identified as major obstacles. Unlike secondary care, where dedicated sustainability roles exist, structured support is very rarely available within general practice.

‘I think the biggest inhibitors are time and money’.

General practitioner

Another major barrier was the impact of leasing versus owning general practice premises. Stakeholders expressed surprise at how these arrangements could restrict the ability to implement sustainable changes, as leased properties often limit the extent to which practices can make structural improvements, such as upgrading insulation or installing renewable energy systems. Stakeholders felt that addressing these barriers requires coordinated efforts involving healthcare commissioners, landlords and general practice teams to establish supportive environments for decarbonisation.

‘I was surprised I had not considered leasing versus owning as impacting general practices’.

PPI representative

### 3.3 Enhancing Public Engagement and Messaging

Stakeholders emphasised the importance of clear, patient‐centred communication in advancing decarbonisation efforts within general practice. Discussions revealed gaps in public understanding of the operational challenges general practices face, such as leasing constraints that limit infrastructure changes.

‘I think that there would be advantages in keeping the public on board. […] It is really important keeping the public interest and involved in what is going on and they maybe, you know, really helpful to implement various sustainability projects’.

PPI representative

Stakeholders discussed concerns about how sustainability actions might inadvertently cause distress or confusion among patients. For example, reducing inappropriate polypharmacy was cited as a decarbonisation strategy, but some stakeholders felt that this could affect trust and be unsettling for patients who have worked with their clinicians to establish a stable medication regimen. Stakeholders stressed the need for carefully crafted and well‐framed messaging that reassures patients about the ongoing prioritisation of their care, while also emphasising the co‐benefits of decarbonisation, such as improved health outcomes.

‘[…] potentially reducing the number of medications people are on. And depending on how we communicate that it could cause some unnecessary distress to those who have fought so hard to get; to find a medication balance that works for them. So, … forward thinking about how we communicate that in a safer, non‐threatening manner, basically’.

PPI representative

Stakeholders emphasised the importance of proactive, solution‐focused messaging that demonstrates the tangible benefits of sustainable practices. They suggested that this should avoid being overly technical and instead focus on co‐benefits that resonate with patients, such as cost‐savings, improved air quality and reduced health inequalities. They also recommended leveraging local community networks, such as climate action groups and patient champions, to disseminate these messages and build grass roots support for sustainability initiatives.

### 3.4 Policy and Leadership as Drivers of Change

Stakeholders stressed the need for Integrated Care Boards (ICBs) to take a more active role in supporting sustainability efforts, as current ICB Green Plans disproportionately focus on secondary care, large buildings and acute hospitals, with insufficient attention given to primary care. This gap in policy has left general practices without sufficient guidance, funding or resources to advance decarbonisation. They stressed the importance of developing green plans that explicitly include primary care and promote shared learning between ICBs to foster consistency and collaboration.

‘[…] if we are talking about strong leadership, ICBs should be actually doing strong leadership in this, which mostly they are not, but that is another source of information for finding out what is going on in ICBs and also ultimately disseminating information and facilitating green plans’.

Policymaker

Financial incentives were identified as powerful tools to drive change in general practice. Stakeholders discussed the success of the Inhaler Impact Framework (IIF) in promoting sustainable prescribing practices and suggested that similar financial mechanisms could be applied to other areas of decarbonisation. They also highlighted the need for clear procurement policies that require supplies of goods and services to demonstrate their own sustainability credentials. For example, current NHS procurement rules mandate that suppliers with contracts over £10,000 annually must have green plans, but this requirement is often overlooked in primary care.

Practice managers were identified as key facilitators of sustainability initiatives. Stakeholders suggested that they should have dedicated time and resources to lead these efforts, as their engagement is crucial for coordinating team‐wide actions and ensuring the successful implementation of green strategies. They also noted the importance of a ‘top‐down’ approach, where national and regional leadership sets clear expectations and provides the necessary support for general practices to integrate sustainability into their daily operations. This is reflective of public organisations' practices centring around targets.

‘One of the other things that struck me was about engaging with practice managers and getting them on board. And it's not something I really thought that much about. But they are really an important stakeholder group in the whole process’.

General practitioner

Key themes and identified needs and actions within each of the themes above are summarised in Table 3.

**Table 3:** Summary of key views.

Views	Main topics and identified needs
Bridging knowledge and resource gaps	− Lack of awareness among GP teams about decarbonisation resources. − Resources are unclear and fragmented, requiring simplification. − Survey response bias suggests lower actual engagement. − Tailored educational materials and proactive dissemination strategies are needed. − Patients are unaware of challenges such as leasing constraints.
Practice‐level barriers to implementation	− Time constraints and heavy workloads limit sustainability efforts. − Few dedicated sustainability roles in general practice. − Organisational culture must embed sustainability as a shared responsibility. − Leasing arrangements restrict structural changes, requiring coordinated support from commissioners and landlords.
Enhancing public engagement and messaging	− Decarbonisation must align with patient‐centred care, addressing concerns such as polypharmacy reduction. − Messaging should be solution‐focused, highlighting co‐benefits such as cost‐savings for the practice and health improvements. − Community engagement via patient champions and local networks is essential.
Policy and leadership as drivers of change	− Primary care is often overlooked in ICB Green Plans. − Commissioners must integrate general practice into decarbonisation policies. − Expanding financial incentives (e.g., IIF) can drive sustainability. − National leadership must provide clear frameworks and resources for practice managers.

*Note:* Within each ICS, Integrated Care Boards (ICBs) are statutory NHS bodies responsible for allocating resources and overseeing service delivery.

The above views capture the perspectives of the participants and underscore the complexity of decarbonisation efforts and the need for coordinated system‐wide approaches to general practice decarbonisation. Although many views were shared across groups, certain emphases differed. For example, public representatives placed stronger focus on patient‐centred communication and transparency; policymakers emphasised system‐level incentives and leadership; and general practitioners highlighted operational constraints. These nuances were integrated below.

#### 3.4.1 Workshop Evaluation

**Figure 1:**
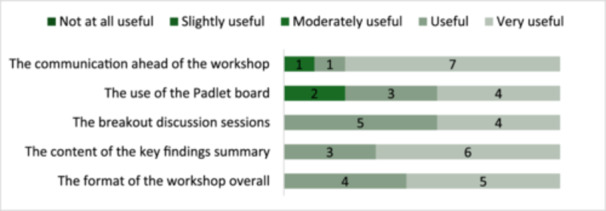
Stakeholder evaluation on the usefulness of several aspects of the KMb workshop.

Overall, stakeholders found the workshop was well‐structured and valuable, with communication and key findings standing out as particularly effective components. The response rate was 60% (*n* = 9/15). Stakeholder feedback (Figure 1) presents consistently positive evaluations across all workshop components, with mean scores ranging from 4.22 to 4.67 on a 5‐point Likert scale (Table 4).

**Table 4:** Summary of workshop evaluation.

Statement	Mean	Standard deviation (SD)
The format of the workshop overall	4.56	0.50
The content of the key findings summary	4.67	0.46
The breakout discussion sessions	4.44	0.49
The use of the Padlet board	4.22	0.76
The communication ahead of the workshop	4.67	0.61

The communication ahead of the KMb workshop (mean = 4.67; SD = 0.61) and the content of the key findings summary (mean = 4.67; SD = 0.46) received the highest ratings, suggesting these were particularly effective aspects. The overall format of the workshop (mean = 4.56; SD = 0.50) and the breakout discussion sessions (mean = 4.44; SD = 0.49) were also rated as highly useful. The use of the Padlet board received slightly more variable feedback (mean = 4.22; SD = 0.76), though still within the ‘useful’ to ‘very useful’ range. All respondents expressed willingness to participate in future in KMb workshops on general practice decarbonisation research (*n* = 9; 100%).

#### 3.4.2 Workshop Impact

The KMb workshop directly informed revisions to both the Policy Brief and Factsheet, ensuring these outputs were clearer, more accessible and tailored to the needs of different stakeholder groups. Feedback from PPI participants highlighted the importance of consolidating and simplifying information using accessible, non‐technical language to enhance public engagement. Stakeholders and commissioners proposed increasing interactivity by embedding links to related resources within the digital materials, thereby improving usability and awareness of the study outputs. They also recommended producing a short 2–3‐min video summarising the key messages from the Factsheet to facilitate wider dissemination through online platforms. GP staff emphasised the need to frame sustainability messages in a way that acknowledges current workload pressures, while reinforcing that sustainable care aligns with good clinical practice. They also advised making the language more inclusive of non‐clinical staff to recognise their contribution to sustainable primary care. These recommendations were subsequently incorporated by the research team following the workshop, enhancing the clarity, accessibility and practical relevance of the Policy Brief and Factsheet, and thereby strengthening their potential for impact and uptake.

## 4 Discussion

The workshop played a crucial role in enriching the interpretation of the research study's key findings by providing multiple and diverse perspectives. It also directly informed the refinement of the study's Policy Brief and Factsheet, enabling these materials to be more carefully tailored to reflect stakeholder information needs, ensuring their relevance for practical application and policy impact. Through the discussions held during this workshop, critical insights were gained into the mobilisation of knowledge to support decarbonisation in general practice. It became evident that while KMb is vital for bridging research, policy and practice it remains constrained by notable gaps in knowledge and information [7]. Participants highlighted the importance of KMb as a facilitator of collaboration, enhancing the relevance and actionability of research findings.

KMb is non‐linear, pragmatic and stakeholder‐driven, with varied knowledge sources, barriers and guideline adoption approaches [12, 23]. It involves the systematic sharing, adaptation and application of research evidence to improve healthcare delivery, patient outcomes and policy implementation [12, 24]. This process requires bridging the gap between research and practice by fostering collaboration among clinicians, policymakers and the public to ensure that knowledge is not only accessible but also actionable [25, 26, 27]. In general practice, KMb has been used to support quality improvement, guideline implementation and service redesign, often requiring tailored approaches that consider the time constraints, resource limitations and diverse needs of general practice settings [12].

Future phases of the GPNET‐0 Study will assess changes in behaviour and decarbonisation actions adoption, including uptake of decarbonisation.

### 4.1 Lessons Learnt

This article underscores the critical role of KMb in advancing decarbonisation efforts within general practice. The findings highlight that while primary care holds significant potential to contribute to the broader net zero healthcare agenda, several barriers currently hinder effective engagement with sustainability initiatives. These barriers include limited awareness, insufficient resources and a lack of organisational support. Additionally, cultural resistance and time constraints further limit the capacity and motivation of general practices to integrate decarbonisation strategies into their routine operations.

To address these challenges, targeted KMb strategies may be needed. These could focus on fostering collaboration between stakeholders, including healthcare professionals, policymakers and local communities, to ensure that decarbonisation efforts are not only practical but also sustainable in the long term. Furthermore, active involvement of policymakers, commissioners and leadership appears crucial in providing clear guidance, financial incentives and adequate resources to support primary care in its decarbonisation journey.

The KMb workshop provided valuable insights that are relevant to future research and policy development. Addressing the identified barriers, particularly by increasing resources and support for general practice, could significantly enhance the uptake of decarbonisation actions. Moving forward, further exploration of the practicalities of implementing these actions across diverse settings, coupled with ongoing engagement with stakeholders, appears likely to be crucial for achieving the broader goal of net zero healthcare.

KMb workshops such as this play a critical role in bridging the gap between research, policy and practice by facilitating meaningful dialogue among key stakeholders. Future iterations could benefit from structured follow‐up mechanisms, such as additional workshops to track progress, sustain engagement and refine implementation strategies based on emerging insights.

### 4.2 Limitations

While the KMb workshop generated important insights, several limitations should be acknowledged. First, the scope of the workshop was constrained to a relatively small number of specific stakeholders. Second, self‐selection bias is likely as participants were generally supportive of the decarbonisation agenda, and opposing views are under‐represented. Third, the findings may have limited transferability to healthcare systems outside England, where organisational and policy structures differ. Fourth, while it was a strength that the KMb workshop was conducted within a 90‐min duration, online to support access and participation with minimal carbon impact, discussions were inevitably time‐bound, potentially restricting deeper exploration. Finally, long‐term impact of the workshop's outputs, such as the Policy Brief and Factsheet, on actual change remains to be evaluated.

Future research should longitudinally assess the sustained impact and cost‐effectiveness of KMb on decarbonisation in general practice, exploring how different dissemination format, stakeholder groups and engagement strategies translate knowledge into measurable and equitable sustainability outcomes.

## 5 Conclusion

This KMb workshop about decarbonisation in general practice has provided valuable insights into the needs of various groups and the most effective ways to disseminate our research findings. These insights have directly informed the planning of study outputs. While this process requires considerable time, resources and careful planning, it is essential for ensuring that dissemination strategies are grounded in a clear understanding of what is truly useful for different stakeholders. Without this engagement, dissemination efforts would lack the needed context and relevance for key audiences.

## Author Contributions

**Ana Raquel Nunes:** funding acquisition (lead), project administration (lead), conceptualisation (lead), methodology (lead), data curation (equal), formal analysis (lead), writing – original draft preparation (lead). **Olivia Geddes:** data curation (equal), writing – review and editing (supporting). **Florence Karaba:** data curation (equal), writing – review and editing (supporting). **Abi Eccles:** data curation (equal), writing – review and editing (supporting). **Helen Twohig:** data curation (equal), writing – review and editing (supporting). **Helen Atherton:** writing – review and editing (supporting). **Frederik Dahlmann:** writing – review and editing (supporting). **Michael Gregg:** writing – review and editing (supporting). **Rachel Spencer:** writing – review and editing (supporting). **Jeremy Dale:** funding acquisition (lead), conceptualisation (lead), methodology (lead), data curation (equal), writing – review and editing (supporting).

## Ethics Statement

This study should be considered as public involvement/contribution in research. The workshop, being stakeholder engagement and not research, did not require ethical approval. Experts by lived experience who took part in our workshop were not research participants.

## Conflicts of Interest

The authors declare no conflicts of interest.

## Supporting information


**Supporting Material 1:** Agenda.

**Supporting Material 2:** Policy Brief Aug 200001.

**Supporting Material 3:** Factsheet Aug 2024.

**Supporting Material 4:** Evaluation Form.

## Data Availability

There is no research data associated with this article. All additional information requests should be submitted to the corresponding author for consideration.
