# Supplementary material for: Mobilising Knowledge for General Practice Decarbonisation: Maximising Impact Through a Multi‐Stakeholder Workshop
Source: Health Expect. 2025 Nov 3;28(6):e70477. doi: 10.1111/hex.70477 (PMC12580982; doi:10.1111/hex.70477)
Supplement: Supplementary file 1 — Supporting Material 1: Agenda. [file HEX-28-e70477-s002.docx]

**Workshop agenda**

11:00 – 11:05 Welcome, introduction, and the purpose of the workshop

11:05 – 11:23 Presentation of key findings, introduce Policy Brief and Knowledge Mobilisation Factsheet

11:25 – 11:35 Small group discussions – Interpreting findings

11:35 – 11:45 Feedback from small groups and plenary discussion

11:45 – 11:50 Break: 3 minutes

11:50 – 11:55 Presentation of current target audiences and dissemination strategies

11:55 – 12:05 Small group discussions – Target audiences and dissemination strategies

12:05 – 12:15 Feedback from small groups and plenary discussion

12:20 – 12:30 Summary and next steps
