# Supplementary material for: Mobilising Knowledge for General Practice Decarbonisation: Maximising Impact Through a Multi‐Stakeholder Workshop
Source: Health Expect. 2025 Nov 3;28(6):e70477. doi: 10.1111/hex.70477 (PMC12580982; doi:10.1111/hex.70477)
Supplement: Supplementary file 2 — Supporting Material 2: Policy Brief Aug 200001. [file HEX-28-e70477-s003.docx]

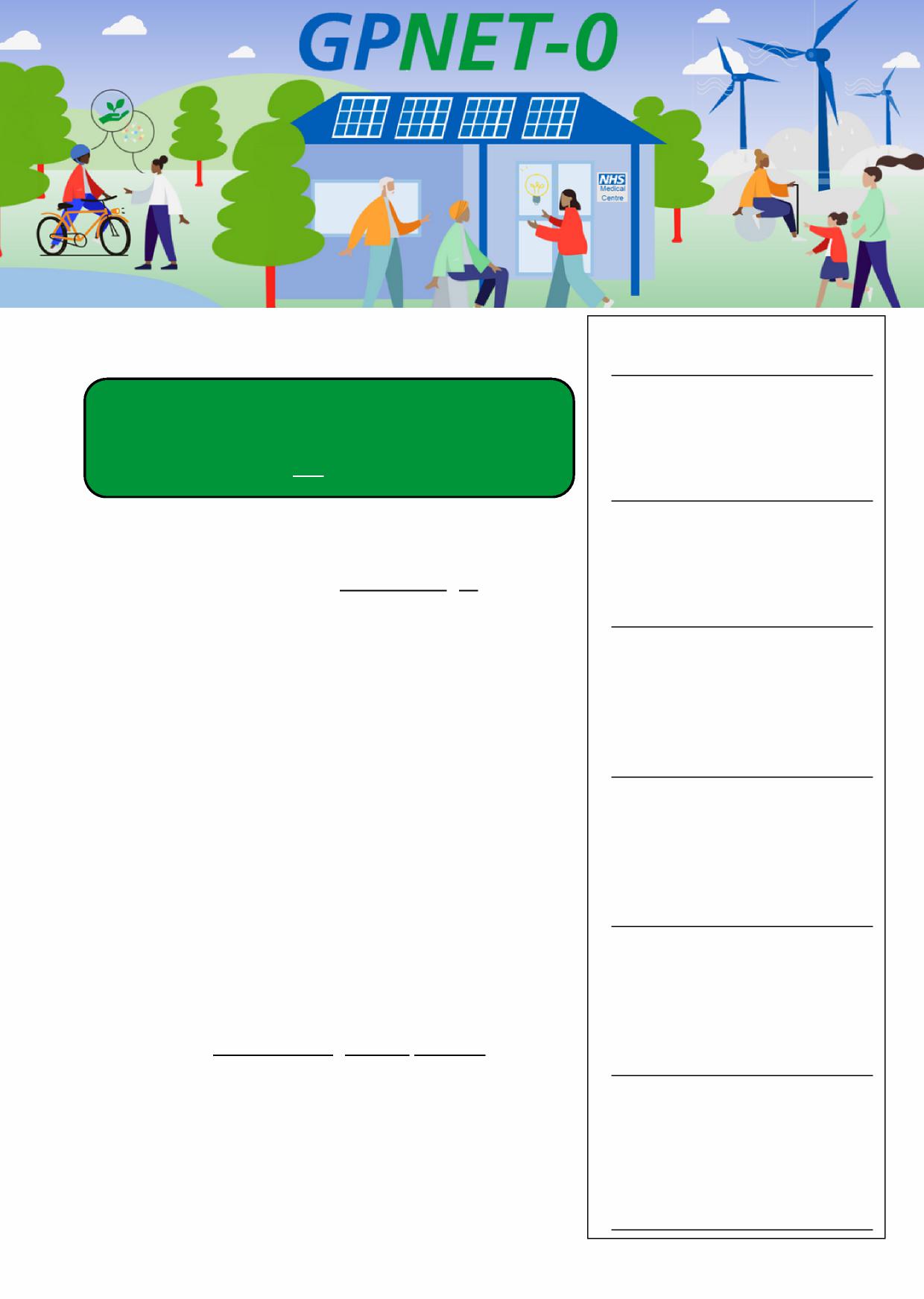


**August 2024 Issue #1**

**Policy brief**

For the NHS to achieve its [net zero target,](https://www.england.nhs.uk/greenernhs/) General Practice will need to accelerate actions aimed at decarbonising its activities.

Little is currently known about how general practices are approaching decarbonisation, including how resources available to support decarbonisation actions are being used.

This is where the GPNET-0 Study contributes:

**First** to understand how general practice is implementing decarbonisation actions to help achieve a net zero NHS.

**Second**, to generate actionable recommendations on how to support and accelerate the implementation and sustainability of actions to [decarbonise](https://seesustainability.co.uk/decarb-gp-guide#:~:text=Decarbonisation%20guide%20for%20General%20Practice%201%20Examines%20the,model%20to%20deliver%20excellent%20primary%20care%20for%20patients.)  [general](https://seesustainability.co.uk/decarb-gp-guide#:~:text=Decarbonisation%20guide%20for%20General%20Practice%201%20Examines%20the,model%20to%20deliver%20excellent%20primary%20care%20for%20patients.)  [practice](https://seesustainability.co.uk/decarb-gp-guide#:~:text=Decarbonisation%20guide%20for%20General%20Practice%201%20Examines%20the,model%20to%20deliver%20excellent%20primary%20care%20for%20patients.) to help achieve a net zero NHS.

1

This policy brief produced by the NIHR-funded GPNET-0

study team is to share key evidence as it emerges. For

information about the study and the work that we are doing,

visit [our](https://warwick.ac.uk/fac/sci/med/research/hscience/apc/qualityandsafety/gpnet-0/) webpage.

**Summary of initial findings**

Costs associated with recycling services may act as a decarbonisation barrier.

Time and administrative constraints impact on the planning and implementation.

The practice’s setting appears to impact the patients’ and staff capacity to use greener modes of active transport

Owning as opposed to leasing premises allows practices to adapt a wider range of decarbonisation initiatives.

Co-benefits such as cost savings and financial incentives are potentially key drivers of decarbonisation action.

Organisational culture may play a key role in shaping decision-making processes related to decarbonisation initiatives.


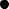


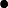


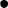


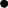


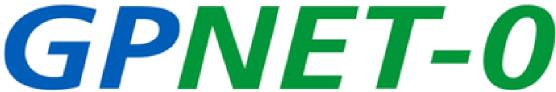


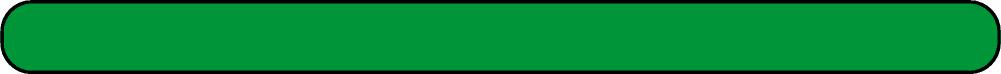


The 30-month GPNET-0 study commenced in September 2023

**Evidence Base**

**Systematic review**

Conducted a systematic review of to identify the key components of processes, behaviours and activities that support decarbonisation in general practice.

15 studies identified, most of which were from the UK (5), Australia (3), and the USA (2), with one each from Germany, France, Switzerland, and Israel.


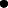


**Findings**

***Knowledge and awareness***: limited access to information and insufficient climate change knowledge among primary care physicians and patients. Patient and community engagement: cooperation between stakeholders and community engagement and support in sustainable healthcare practices as well as a patient-centred approach were identified as facilitating the implementation of sustainability initiatives.

***Practice management and leadership***: decarbonisation actions initiated solely by staff without management involvement were observed to be implemented less consistently. Leadership that prioritizes environmental sustainability was identified as crucial in driving change by influencing staff engagement and workplace culture.

***Personal and professional integration***: climate change initiatives were perceived as time-consuming requiring personal effort, behavioural change and financial investments for practices dealing with increased workloads and staff shortages. However, behavioural adaptations as well as contextual factors including financial incentives, centralisation of infrastructure and services and political action served as facilitative elements.

2


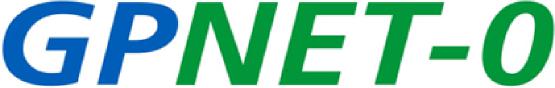


**General Practice Survey**

3

The survey received 328 responses from 163 practices (34% of total).


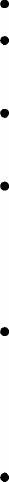


GP Partners made up the highest proportion of respondents (29.9%), followed by those in managerial roles (23.3%) and those in administrative roles (18.6%). 54 (33.1%) practices had more than one respondent, with the total number of respondents per practice varying from 2 to 23.

General practice staff showed an overall ***willingness*** to undertake

decarbonisation actions. 69.8% of respondents agreed that acting to reduce greenhouse gas emissions from primary care was a legitimate part of their role.

Respondents showed ***general unawareness*** of the resources currently available to general practice teams to support decarbonisation initiatives. This was reflected when respondents were asked to indicate their awareness of 10 decarbonisation resources (see figure 1).

There was a ***disparity in understanding*** of decarbonisation activities across multiple respondents from the same practice. A third of respondents answered ‘yes’ when asked if their practice had a lead for decarbonisation, equivalent to 35.6% of responding practices. The most highly cited job role of the individual in this lead position was a GP Partner (46.7%). 31% of practices with a decarbonisation lead had this reported by multiple respondents, however in some instances, there was disparity in the reporting of the role of the lead individual from members of the same practice. This suggests challenges in information sharing and a lack of strong leadership regarding decarbonisation.

**Figure 1**: Practice level staff awareness of decarbonisation resources available to general practice


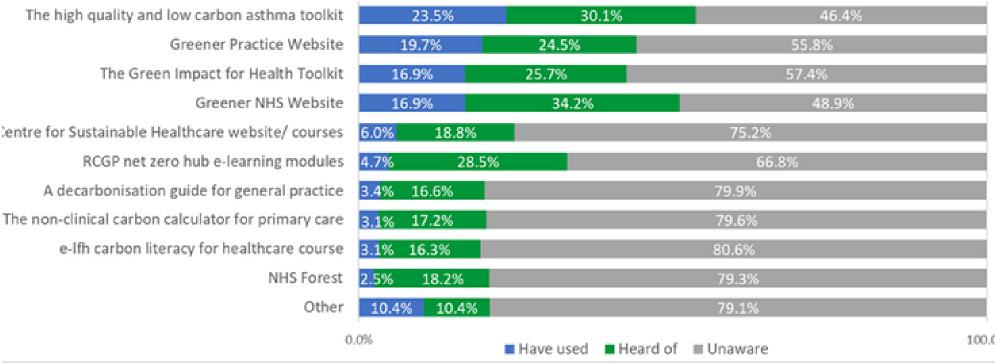


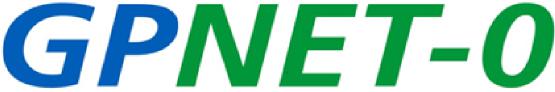


**General practice case studies**

Based on the preliminary observations during baseline workshops - the following factors are emerging as influential in the planning and implementation of decarbonisation actions within the 12 case study sites enrolled in the longitudinal study.

**Initial findings**

***Organisational culture***: a hierarchical organisational culture emerged as an influential factor during meetings with practices. For example, during the baseline workshops, clinicians were more likely to engage in discussions about potential decarbonisation measures than non-clinicians. This could be an indication that any initiatives adopted by a practice required decision-making at the practice management level because of the resulting financial decisions. ***Non-clinical carbon footprint***: prioritising [non-clinical](https://seesustainability.co.uk/carbon-footprint) over clinical carbon reduction was cited as easier to implement as the practice had more autonomy over the selected actions.

***Available resources***: staff were familiar with some decarbonisation resources more than others. For example, a wider staff awareness of the [Asthma toolkit](https://www.greenerpractice.co.uk/high-quality-and-low-carbon-asthma-care/) was observed which may be a reflection of the impact of an NHS initiative to reward PCNs for switching patients to salbutamol MDIs with a lower carbon intensity propellant (Robinson, 2021).

***Concomitant benefits***: co-benefits of decarbonisation initiatives such as cost savings, financial incentives, and CQC compliance were deemed important by several practices. For instance, several practices with high energy bills noted their interest in pursuing decarbonisation measures in order to [reduce their](https://www.sseenergysolutions.co.uk/small-business-sustainability/business-energy-audits)  [energy costs](https://www.sseenergysolutions.co.uk/small-business-sustainability/business-energy-audits).

***Leased versus owned practice premises***: Practices that lease their premises face limitations on the decarbonisation measures they can implement, especially regarding energy usage and building services.

***The practice’s setting*** (e.g. rural vs urban)may impact patients’ and staff capacity to use [active modes of transportation](https://elearning.rcgp.org.uk/mod/page/view.php?id=12583).

***Waste and recycling***: High recycling costs pose a barrier to practices’ efforts to reduce their carbon footprint. Some practices reported that staff resorted to taking recyclable waste home to dispose of as they lacked a recycling contract with a commercial contractor (local councils waste collections are only for domestic waste).


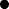


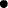


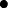


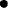


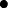


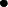


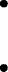


4


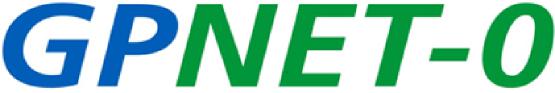


**Initial findings continued**


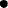


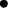


***Role of champions***: Climate change champions can have a mixed effect on a practice’s decarbonisation efforts. While they inspire co-workers about climate change, their commitment may inadvertently deter full engagement from staff who might assume that the champions alone are responsible for all sustainability actions within the practice.

[***Time and administrative***](https://toolkit.sos-uk.org/greenimpact/giforhealth/login) ***constraints***: these limitations were identified by some practices as key factors with an impact on the planning and implementation of decarbonisation actions. This was, for example, the case at a small practice with three GPs and where all staff worked part-time. They expressed concern over the study’s timeframe, saying that conflicting tasks and priorities may come up for team members potentially interfering with the implementation of decarbonisation activities.

**Acknowledgements**

This report presents independent research funded by the National Institute for Health and Care Research ([NIHR](https://www.nihr.ac.uk/)).

5

**Contact details**

GPNET-0 Study

Warwick Medical School

Webpage click [here](https://warwick.ac.uk/fac/sci/med/research/hscience/apc/qualityandsafety/gpnet-0/).
