# Supplementary material for: Mobilising Knowledge for General Practice Decarbonisation: Maximising Impact Through a Multi‐Stakeholder Workshop
Source: Health Expect. 2025 Nov 3;28(6):e70477. doi: 10.1111/hex.70477 (PMC12580982; doi:10.1111/hex.70477)
Supplement: Supplementary file 3 — Supporting Material 3: Factsheet Aug 2024. [file HEX-28-e70477-s004.docx]

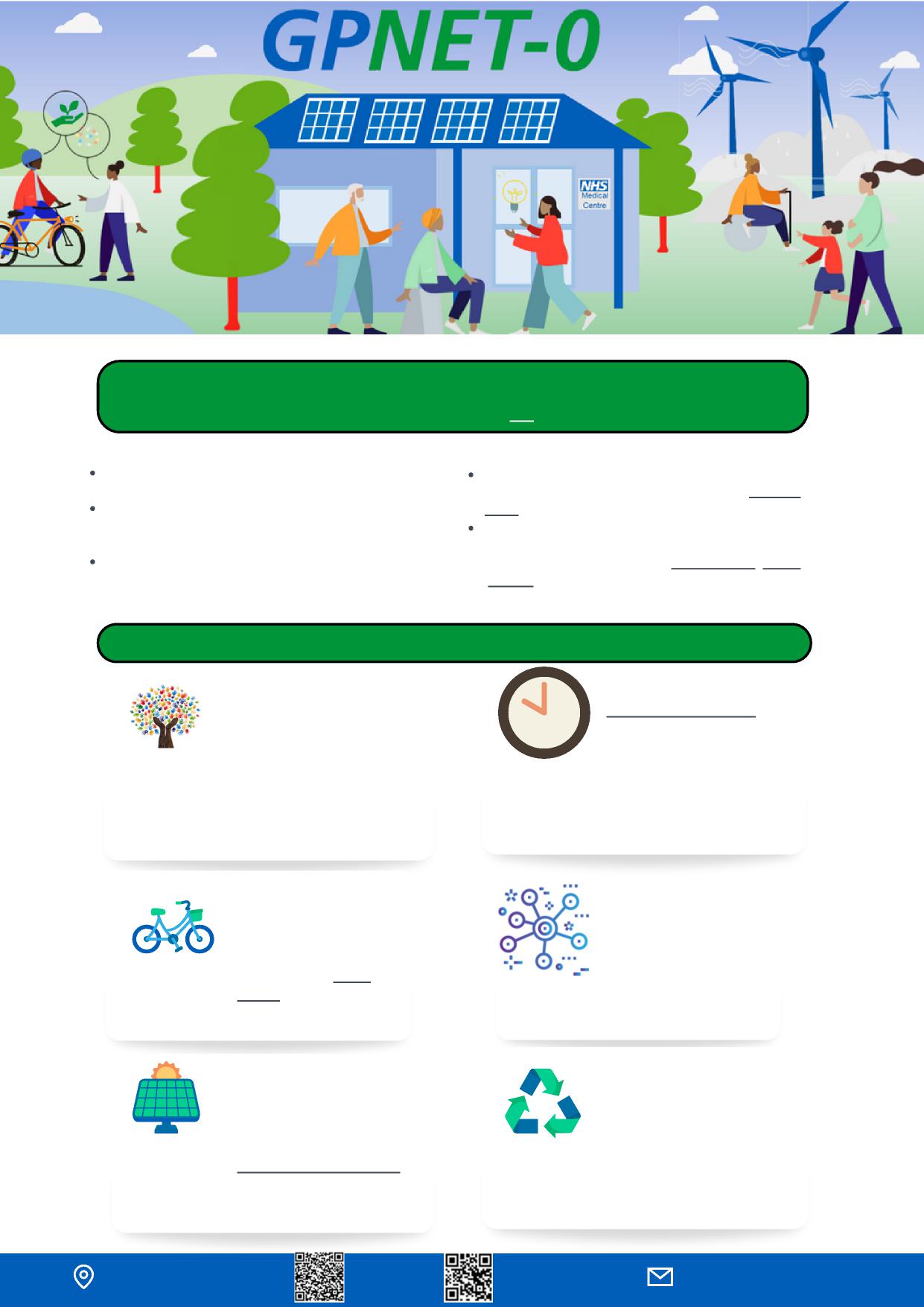


The practice’s setting may impact patients’ and staff capacity to use [active](https://elearning.rcgp.org.uk/mod/page/view.php?id=12583)  [modes](https://elearning.rcgp.org.uk/mod/page/view.php?id=12583) of transportation.

**03**

**FACTSHEET #1 AUGUST 2024**

This factsheet produced by the NIHR-funded GPNET-0 study team is to share key evidence as it emerges. It

includes links to online resources that are relevant to the issue concerned. For information about the study and

the work that we are doing, visit [our](https://warwick.ac.uk/fac/sci/med/research/hscience/apc/qualityandsafety/gpnet-0/)  webpage.

**NHS and Climate change**

The NHS contributes 25% to all public sector carbon emissions.

Primary care is responsible for around 23% of NHS emissions through direct care delivery, staff and patient travel, and other related services.

The NHS is the world's first health service to commit to reaching carbon net zero.

**GPNET-0 study aims**

To understand how general practice is implementing decarbonisation actions to help achieve a [net zero NHS.](https://www.england.nhs.uk/greenernhs/)

To generate actionable recommendations on how to support and accelerate the implementation and sustainability of actions to [decarbonise](https://seesustainability.co.uk/decarb-gp-guide#:~:text=Decarbonisation%20guide%20for%20General%20Practice%201%20Examines%20the,model%20to%20deliver%20excellent%20primary%20care%20for%20patients.)  [general practice](https://seesustainability.co.uk/decarb-gp-guide#:~:text=Decarbonisation%20guide%20for%20General%20Practice%201%20Examines%20the,model%20to%20deliver%20excellent%20primary%20care%20for%20patients.) to help achieve a net zero NHS.

**Initial findings**

**Time**

**Organisational culture**

Organisational culture may play a key role in shaping decision-making processes related to decarbonisation initiatives.

[Time and administrative](https://toolkit.sos-uk.org/greenimpact/giforhealth/login) constraints impact on the planning and implementation of decarbonisation actions.

**02**

**01**

Key issues that are emerging as influencing how general practice is addressing the need to decarbonise include:

**Practice setting**

**Leasing vs owning**

Owning as opposed to leasing premises allows practices to adapt a wider range of [decarbonisation initiatives](https://seesustainability.co.uk/decarb-gp-guide#:~:text=Decarbonisation%20guide%20for%20General%20Practice%201%20Examines%20the,model%20to%20deliver%20excellent%20primary%20care%20for%20patients.).

**05**

Costs associated with recycling services may act as a barrier.

**06**

**Co-benefits**

Co-benefits of initiatives such as cost savings and financial incentives are potentially key drivers of decarbonisation action.

**04**

**Recycling**

Sign up to our

Warwick Medical School Website newsletter

[gpnet0@warwick.ac.uk](mailto:gpnet0@warwick.ac.uk)

**1**


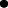


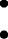


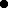


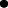


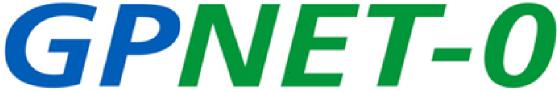


EVIDENCE BASE

**Systematic review**

To identify the key components of processes, behaviours and activities that support decarbonisation in general practice.

15 studies identified, most of which were from the UK (5), Australia (3), and the USA (2), with one each from Germany, France, Switzerland, and Israel.


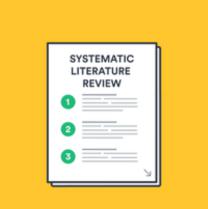


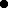


**General practice survey**

To measure current interest and involvement in decarbonisation activities in general practice, we have surveyed general practice staff in 3 ICB areas (Coventry and Warwickshire; Birmingham and Solihull; South Yorkshire); there were 328 responses from 163 practices (34% of total).

The key findings were:

69.8% staff agreed that acting to reduce carbon emissions from primary care was a legitimate part of their role.

51.2% staff were unaware of any new or additional decarbonisation actions that their general practice is considering.

86.6% staff felt that they lacked sufficient training and resources to undertake decarbonisation initiatives.


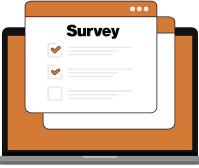


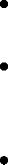


**12-month longitudinal study**

12 practices with varied characteristics are being followed over 12 months to learn how they plan and undertake decarbonisation activities.

4 practices per ICB

The practices have a range of characteristics: list size and number of GPs in the practice, level of deprivation, % of patients from an ethnic minority group, location (rural, semi-rural, urban), practice engagement with decarbonisation activities (low, medium, high), geographical area. So far, we have facilitated each practice to produce a Green Action Plan setting that reflects their own ambitions, and to undertake baseline non-clinical carbon calculations. We are meeting with each practice on a 3-monthly basis to discover the progress that they have made with implementing their Green Action Plan and to understand what has influenced this.


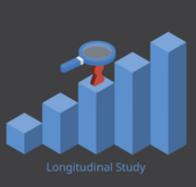


For more information about the GPNET-0 study, visit the website [here](https://warwick.ac.uk/fac/sci/med/research/hscience/apc/qualityandsafety/gpnet-0/).

2
