# Supplementary material for: Mobilising Knowledge for General Practice Decarbonisation: Maximising Impact Through a Multi‐Stakeholder Workshop
Source: Health Expect. 2025 Nov 3;28(6):e70477. doi: 10.1111/hex.70477 (PMC12580982; doi:10.1111/hex.70477)
Supplement: Supplementary file 4 — Supporting Material 4: Evaluation Form. [file HEX-28-e70477-s001.docx]

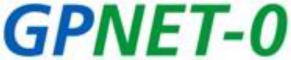


**Thank you for attending the 1st GPNET-0 Study Knowledge

Mobilisation Workshop.**

We would really appreciate you taking a few minutes to anonymously

share your feedback to help us plan future workshops.

*Q1. Please select what best describes the capacity in which you attended the GPNET-0 Study Knowledge Mobilisation Workshop (select all that apply).

Public patient involvement (PPI) member

General practice stakeholder

NHS/primary care sustainability champion

Policymaker

Commissioner

Other (please specify)

*Q2. On a scale of 1-5 (1 'not at all useful' to 5 'very useful'), how useful did you find...

1 2 3 4 5

The format of the workshop overall


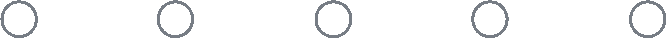


The content of the key findings summary


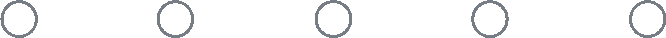


The breakout discussion sessions


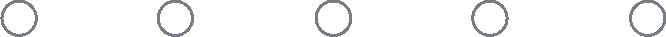


The use of the padlet board


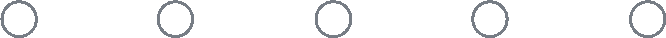


The communication ahead of the workshop


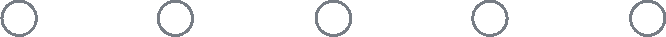


Q3. Was there any additional information you would have liked to have received ahead of, or during the workshop?

Yes (add details)

No


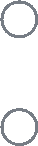


Q4. How could we improve future GPNET-0 Study Knowledge Mobilisation Workshops?

[Powered by Qualtrics](https://www.qualtrics.com/powered-by-qualtrics/?utm_source=internal%2Binitiatives&utm_medium=survey%2Bpowered%2Bby%2Bqualtrics&utm_content=warwick&utm_survey_id=SV_1ETEufz846v178O)

Protected by reCAPTCHA: [Privacy](https://policies.google.com/privacy?hl=en) & [Terms](https://policies.google.com/terms?hl=en)


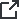


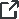


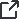


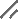


Q5. Do you have any suggestions regarding other

individuals/organisations who it would be valuable to invite to future GPNET-0 Study Knowledge Mobilisation Workshops?


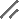


*Q6. Based on your experience today, would you like to take part in future GPNET-0 Study knowledge mobilisation workshops?

| 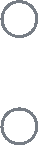 | Yes please  No, thank you (add details) |
| --- | --- |

Next page
